# Immune-Cell-Based Therapy for COVID-19: Current Status

**DOI:** 10.3390/v15112148

**Published:** 2023-10-25

**Authors:** Yiyuan Wang, Qinghe Liang, Fengsheng Chen, Jiehuang Zheng, Yan Chen, Ziye Chen, Ruopeng Li, Xiaojuan Li

**Affiliations:** 1 Laboratory of Anti-Inflammatory and Immunomodulatory Pharmacology, Innovation Program of Drug Research on Inflammatory and Immune Diseases, Southern Medical University, Guangzhou 510515, China; wyy1107@smu.edu.cn (Y.W.); qh_liang_smu@163.com (Q.L.); 18224002562@163.com (F.C.); zhengjiehuang01@163.com (J.Z.); chenyan2652@126.com (Y.C.); 13192672557@163.com (Z.C.); lrp990330@163.com (R.L.); 2 NMPA Key Laboratory for Research and Evaluation of Drug Metabolism, School of Pharmaceutical Sciences, Southern Medical University, Guangzhou 510515, China; 3 Guangdong Provincial Key Laboratory of New Drug Screening, School of Pharmaceutical Sciences, Southern Medical University, Guangzhou 510515, China

**Keywords:** SARS-CoV-2, COVID-19, cell therapy, immunotherapy

## Abstract

Coronavirus disease 2019 (COVID-19), caused by severe acute respiratory syndrome coronavirus 2 (SARS-CoV-2), has become a global pandemic. The interplay between innate and adaptive immune responses plays a crucial role in managing COVID-19. Cell therapy has recently emerged as a promising strategy to modulate the immune system, offering immense potential for the treatment of COVID-19 due to its customizability and regenerative capabilities. This review provides an overview of the various subsets of immune cell subsets implicated in the pathogenesis of COVID-19 and a comprehensive summary of the current status of immune cell therapy in COVID-19 treatment.

## 1. Introduction

The COVID-19 pandemic, caused by the severe acute respiratory syndrome coronavirus 2 (SARS-CoV-2), has had a devastating impact worldwide for over four years. This novel infectious disease has resulted in millions of fatalities, with the death tolls continuing to rise due to the emergence of new variants [1]. COVID-19 presents with respiratory symptoms associated with viral replication, along with systemic effects, making it a complex illness [2]. Common symptoms include fever, cough, and respiratory distress, with severe cases progressing to respiratory failure and even death [3].

The pathophysiology of COVID-19 is primarily characterized by dysregulated immune responses against SARS-CoV-2, involving both innate and adaptive immune components [4]. This dysregulation not only hinders viral clearance but also leads to inflammation, tissue damage in the lungs, and multi-organ damage systemically [5]. Various immune cells, including T cells, natural killer (NK) cells, dendritic cells (DCs), and macrophages, play crucial roles in SARS-CoV-2 infections [6]. Hence, immunotherapy targeting these immune cells presents a promising avenue for mitigating the pathology of SARS-CoV-2 infection, preventing long-term complications, and improving overall survival rates in infectious diseases.

Cellular therapy, an innovative approach to disease treatment, involves the utilization of specialized cells with specific functions obtained through bioengineering and ex vivo expansion, followed by reinfusion into the patient’s body [7]. Chimeric antigen receptor (CAR) T-cell therapy, which has gained approval from the US Food and Drug Administration, has demonstrated significant advancements in the treatment of B-cell malignancies [8]. Immune-cell-based therapies offer distinct advantages over conventional treatments, including high selectivity, localized concentration, and personalization [9]. Over the past decade, cancer immunotherapy has witnessed remarkable success, establishing immune cell therapy as a transformative treatment modality for various diseases, including COVID-19.

In the context of COVID-19, immune cell therapy is a promising approach for effective treatment. This review provides an overview of the different immune cell subsets involved in the pathogenesis of COVID-19 and summarizes the progress in immune cell therapy research and clinical trials targeting COVID-19 (Table 1).

## 2. T Cell Therapy

T lymphocytes, derived from lymphoid stem cells in the bone marrow, undergo differentiation and maturation in the thymus. They then circulate through the lymph and blood, playing a critical role in cellular immunity throughout various immune organs and tissues in the body. Pathological excessive inflammation, associated with lymphocyte reduction and dysregulated T cell responses, is one of the immunological characteristics of COVID-19 [10]. The success of T cell therapy in treating life-threatening viral infections supports its potential application in COVID-19 treatment [11].

### 2.1. Specific T Cell Therapy in COVID-19

**Figure 1:**
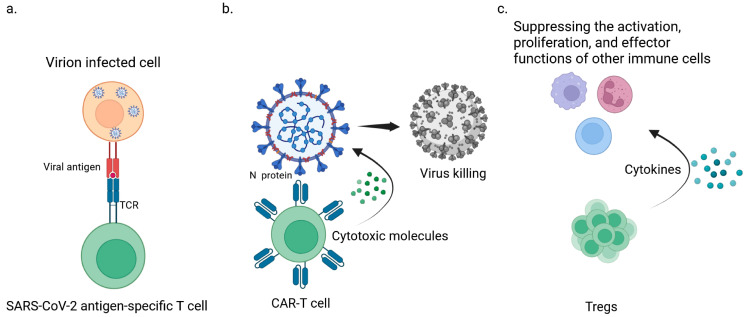
Three types of T cell therapies available for treating life-threatening viral infections. (**a**) SARS-CoV-2-specific T cells that specifically recognize the structural antigen membrane protein of SARS-CoV-2. (**b**). CAR-T cells specially designed to identify the viral antigen N protein of SARS-CoV-2. (**c**). Tregs play a key role in suppressing the activation, proliferation, and effector functions of other immune cells, thereby playing a protective role in inhibiting autoimmune inflammatory reactions in COVID-19 patients with excessive inflammation and cytokine storms.

Specific T cell therapy involves isolating and expanding virus-specific T cells that can recognize the structural proteins of SARS-CoV-2, particularly the membrane protein (Figure 1a). Notably, blood from donors exposed to SARS-CoV-2 contains memory CD4 and CD8+ T cells capable of recognizing various viral antigens, including spike proteins, nucleocapsid proteins, and membrane antigens [12]. Cultivating SARS-CoV-2-specific T cells in the presence of interleukin (IL)-2/4/7 can increase their proliferation by over 1000 times while preserving their phenotype, function, and antigen recognition hierarchy compared to baseline samples [13]. This approach allows for the generation of expanded cytotoxic T lymphocytes (CTLs) targeting the structural proteins of SARS-CoV-2, including the receptor-binding domain of the spike protein [13]. Incorporating HLA-E-restricted CD8 T cells into T cell immunotherapy for COVID-19 offers several advantages, including the ability to eliminate infected cells, inhibit intracellular infection, reduce inflammatory reactions, and limit tissue damage, which are important mechanisms in the pathogenesis of COVID-19 [14].

In vitro experiments have demonstrated that clinically relevant levels of SARS-CoV-2-specific T cells maintain their functionality and proliferative capacity while retaining their specific cytotoxic potential. These T cells exhibit functional and phenotypic stability within days after enrichment [15]. In vitro expansion and isolation of SARS-CoV-2-specific T cells producing IFNγ have been successfully achieved, showing peptide-specific cytolytic and proliferative responses upon re-exposure to the antigen [16]. Furthermore, in vitro expanded SARS-CoV-2 antigen-specific T cells using partially HLA-matched third-party products have shown potential in the treatment of severe cases of COVID-19 unresponsive to previous interventions [17]. Currently, a phase I clinical trial is underway to assess the safety and feasibility of immunotherapy with infused CD45RA memory T cells, including SARS-CoV-2-specific T cells, for the treatment of moderate to severe COVID-19 cases [18]. Additionally, a randomized (2:1), open-label phase I/II trial is evaluating the safety and efficacy of pre-made, partially human leukocyte antigen (HLA)-matched SARS-CoV-2-specific T cells from recovered patients (CoV-2-STs) in combination with standard-of-care therapy (SoC) for the treatment of severe COVID-19 patients [19]. Numerous clinical trials investigating specific T cell therapies for COVID-19 are currently underway (Table 1).

### 2.2. CAR-T Cell Therapy in COVID-19

CAR-T cells, which are engineered T cells with the ability to specifically recognize and attack tumor cells, have emerged as a promising approach for combating COVID-19. These CAR-T cells are designed to target SARS-CoV-2 viral antigens, particularly the N protein(Figure 1b). Mathematical models have been developed to describe the dynamics of infection involving the virus, CAR-T cells, and memory cells. Theoretical analysis suggests that this approach can generate positive therapeutic effects by delaying viral production, which is particularly beneficial in the early stages of infection. This study highlights the potential use of CAR-T cells as an antiviral therapy for COVID-19 [20].

## 3. Tregs Therapy

Regulatory T cells (Tregs) are a group of immune-suppressive cells that play a crucial role in maintaining immune homeostasis and preventing autoimmune reactions [21,22]. Some studies have indicated a decrease in the number of Tregs in the peripheral blood of COVID-19 patients [23,24,25,26]. Mahmoud et al. evaluated the frequencies of Tregs and Th17 cells in healthy individuals and COVID-19 patients using flow cytometry. The results showed a significant decrease in the frequency of Tregs and an increase in the frequency of Th17 cells and the Th17/Treg ratio, particularly in severe COVID-19 patients. Moreover, the expression levels of ROR-γt and FoxP3 were found to increase and decrease, respectively, as the disease progressed [24]. Another study demonstrated that non-survivor patients had lower levels of Tregs and lower FOXP3 expression compared to survivors. However, conflicting results regarding changes in Treg levels in COVID-19 patients have also been reported. Some studies have shown a significant increase in the frequency of CD25^+^CD127^-^Foxp3^+^ Tregs in critically ill COVID-19 patients [27,28].

However, Tregs play a crucial role in suppressing the activation, proliferation, and effector functions of other immune cells, and they are involved in inhibiting self-immune inflammatory reactions. Tregs may exert a protective effect on COVID-19 patients with excessive inflammation and cytokine storms [29,30]. Based on the anti-inflammatory characteristics of Tregs, Treg-based immunotherapy may provide a viable option for treating COVID-19 (Figure 1c). This viewpoint is supported by a recent case study that examined the effects of Treg transplantation from umbilical cord blood. Two patients with acute respiratory distress syndrome received two to three different allogeneic umbilical-cord-blood-derived Tregs that were expanded in vitro, resulting in a significant reduction in the production of inflammatory mediators and achieving good efficacy. Further clinical trials are being conducted to explore the use of umbilical-cord-blood-derived Tregs for the treatment of COVID-19-related acute respiratory distress syndrome (ARDS) (Table 1) [31]. Additionally, Harb et al. found that increased expression of Notch4 in circulating Tregs is associated with disease severity and can predict mortality, with decreased expression observed after recovery. Increased Notch expression can restrict Treg-mediated tissue repair and promote severe lung inflammation during viral infections [32]. Inhibiting Notch expression may be a potential approach for treating COVID-19. Currently, there are four clinical trials for Treg cell therapy in COVID-19, including two completed and two terminated (Table 1).

## 4. NK Cell Therapy

NK cells, also known as natural killer cells, are a type of large granular lymphocyte belonging to the innate immune system. They constitute approximately 5–20% of the total lymphocyte population in the human body [33]. NK cells originate from lymphoid progenitor cells and undergo maturation at various locations, such as the bone marrow, thymus, and spleen, before entering the bloodstream [34]. In response to viral infection, NK cells can directly or indirectly eliminate virus-infected cells through the release of perforin and granzymes or by exerting antibody-dependent cellular cytotoxicity (ADCC) [35]. Cytokines like IL-2, IL-12, and IL-15 can induce NK cells to secrete pro-inflammatory cytokines, such as TNF-α and IFN-γ, activating other immune responses to control viral infections [36]. Notably, COVID-19 patients often experience a significant decrease in NK cell numbers [37].

Building upon the understanding of NK cell biology, many researchers have utilized NK cell therapy to treat viral infections caused by key human pathogens, including SADS, influenza, and the SARS-CoV-2 virus [38,39,40]. NK cell therapy has demonstrated promising results in the treatment of COVID-19 infection in animal studies. Lu et al. developed mACE2-CAR, a fusion protein combining a mutated fragment of ACE2 (mACE2) with intracellular signaling domains CD28 and CD3ζ, and incorporated it into NK cells isolated from umbilical cord blood, resulting in mACE2-CAR-sIL15 NK cells. The study revealed that these NK cells exhibited strong protective effects against SARS-CoV-2 infection in K18-hACE2 mice, a transgenic mouse model expressing human ACE2 in respiratory epithelial cells [41].

NK cell therapy for the treatment of COVID-19 has progressed to the clinical trial stage (Table 1). CAR-NK cells, derived from umbilical cord blood, have been genetically engineered to target two specific molecules, MKG2D and ACE2. By binding to these molecules, CAR-NK cells can prevent SARS-CoV-2 viral infection of ACE2-expressing host cells and enhance the cytotoxicity of CAR-NK cells, enabling rapid elimination of virus-infected cells [42]. Liu et al. constructed and prepared universal IL15 super-antagonist and GM-CSF neutralizing scFv-secretory NKG2D-ACE2 CAR-NK cells from umbilical cord blood. These CAR-NK cells target the S protein of SARS-CoV-2 and NKG2DL on the surface of infected cells while also providing a preventive effect against cytokine release syndrome (CRS) through IL15 super-antagonist and GM-CSF neutralizing scFv. This approach facilitates viral particle clearance. Furthermore, ACE2 CAR-NK cells can competitively inhibit SARS-CoV-2 infection of type II alveolar epithelial cells and other vital organs or tissues, effectively terminating the SARS-CoV-2 infection. That study is currently in phase 1/2 of clinical trials [43]. Another approach, known as CYNK-001 therapy, utilizes non-genetically-modified natural killer (NK) cells derived from cryopreserved human placental hematopoietic stem cells. CYNK-001 cells are obtained by expanding and differentiating placental hematopoietic stem/progenitor CD34^+^ cells through a 35-day culture process [44]. CYNK-001 cells express NKG2D and CD94, as well as NK activation receptors DNAM1, NKp30, NKp46, and NKp44. In preclinical studies, CYNK-001 has demonstrated a range of biological activities expected from NK cells, including perforin and granzyme B expression. Currently, CYNK-001 is undergoing phase 1/2 clinical trials for the treatment of COVID-19 infection [45].

## 5. DC Cell Therapy

Dendritic cell (DC) therapy has emerged as a promising approach in the field of immunotherapy. DCs are considered the most effective antigen-presenting cells and play crucial roles in both innate and adaptive immunity, as well as in the induction of immune tolerance [46]. When exposed to viruses, DCs undergo stimulation and differentiate into two distinct types. Uninfected DCs exert antiviral immune functions, while infected DCs participate in immune evasion processes [47]. SARS-CoV-2 has the ability to infect DCs, and studies have shown that this infection can lead to a decrease in the number and function of DCs, resulting in acute immune dysfunction [48]. This immune dysfunction contributes to the immune evasion of SARS-CoV-2.

Current research has identified various mechanisms through which SARS-CoV-2 can invade host cells, including binding with angiotensin-converting enzyme 2 (ACE2), interacting with DC-specific intercellular adhesion molecule-3-grabbing non-integrin (DC-SIGN), interacting with CD147, and interacting with the dipeptidyl peptidase-4 (DPP4) receptor [49,50]. DC-SIGN and CD147 are expressed on DCs, while ACE2 is expressed on pulmonary interstitial DCs. Studies suggest that SARS-CoV-2 can replicate within DCs, although DCs do not transmit the newly generated virus, indicating the ability of SARS-CoV-2 to infect DCs. Clinical data further indicate a decrease in the proportion of conventional DCs (cDCs) and plasmacytoid DCs (pDCs) during the acute and recovery phases of the disease [48], as well as a reduction in the expression of IFN-α, IFN-β [51,52], and co-stimulatory molecules. The impaired and disrupted function of DCs hampers innate and adaptive immune responses, aiding in the viral escape from the host’s immune mechanisms [53].

Currently, COVID-19 vaccine development primarily focuses on viral vectors, nucleic acids (RNA), protein subunits, and inactivated or killed viruses. However, DCs have emerged as potential candidates for vaccine development, as they can process antigens and present them to immune cells. Clinical trials are currently underway to explore the application of DCs in generating COVID-19 vaccines (Table 1). Strategies for vaccine preparation based on DCs include the adoption of chimeric antigen receptor (CAR) strategies, in vitro loading of autologous DCs with SARS-CoV-2 S protein, combining DCs with nanotechnology, and targeting vaccine antigens to DCs through surface receptors, among others [54]. Currently, there are a limited number of vaccines based on DC or DC-enhancing strategies, as well as nanoparticle (NP)-based vaccines to enhance DC function against SARS-CoV-2, with approximately five in total. The highest clinical stage reached is phase III [55]. Developing vaccines that target the role of DCs in immunity offers unique advantages due to the pivotal role of DCs in innate and adaptive immunity, as well as their susceptibility during infection. Vaccination targeting DCs has the potential to address defects in innate and adaptive immune responses, leading to improved outcomes and contributing to long-term and widespread immunity.

## 6. Monocyte–Macrophage Cell Therapy

The monocyte–macrophage system consists of monocytes in the bloodstream and tissue-resident macrophages [56]. In the lung tissue of mild COVID-19 patients, the number of monocyte–macrophages is low [57]. However, in severe COVID-19 patients, the infection of alveolar epithelial cells by SARS-CoV-2 induces the production of chemokine CCL2, which attracts a large number of circulating monocytes and tissue-resident alveolar macrophages to the site of infection [58]. Once in the tissue, monocytes enhance their phagocytic capacity and gradually differentiate into macrophages, which further secrete pro-inflammatory cytokines (IL-6, IL-7, TNF, etc.) and chemokines (CCL2, CCL3, CCL7, CCL10, etc.), mediating immune responses and engulfing pathogens [59]. It is noteworthy that the substantial amount of cytokines produced by these macrophages in the lung tissue of severe patients can promote pulmonary fibrosis, which is a significant cause of death in COVID-19 patients [60].

Although monocyte–macrophages have long been implicated in promoting tissue fibrosis, due to the plasticity of monocyte–macrophages’ function, recent studies have shown that they also play a pivotal role in fibrosis regression, in part through the expression of matrix-degrading metalloproteinase enzymes (MMPs) [61]. Thus, increasing anti-fibrotic monocyte/macrophage populations is important for clearing partially degraded collagen fragments of the extracellular matrix, especially fibrillar collagen. An attractive therapeutic strategy would be the use of monocytes modified in vitro as a cell therapy to induce fibrosis regression. MON002 is an autologous monocyte cell product that undergoes ex vivo culture before being intravenously injected into patients with post-COVID-19 pulmonary fibrosis [20]. The MONACO Cell Therapy Study is a prospective, non-randomized, open-label phase I/II clinical trial aimed at evaluating the safety of MON002 in five adult patients diagnosed with interstitial lung disease (pulmonary fibrosis) recovering from acute COVID-19 infection (Table 1).

## 7. MSC Therapy

Mesenchymal stem cells (MSCs) are a type of adult stem cell that originate from the mesoderm and have the potential for self-renewal and multidirectional differentiation. They can differentiate into various mesenchymal tissues, such as bone, cartilage, adipose tissue, and bone marrow hematopoietic tissue. During the process of COVID-19 infection, MSCs reduce cytokine storms, limit organ inflammation and fibrotic damage, and regulate innate and adaptive immune responses through the release of bioactive molecules and direct cell–cell contact. Additionally, MSCs can modulate the clearance of alveolar fluid and the permeability of pulmonary endothelial cells, thereby improving lung injury and associated inflammatory reactions. It is worth noting that most MSCs do not express the ACE2 receptor, and, after intravenous injection, MSCs are distributed predominantly in the pulmonary microvasculature, enhancing the persistence of their effects through this unique immune evasion mechanism, making them potential candidates for COVID-19 treatment [62,63]. The safety of MSC-based therapeutic approaches has been confirmed in various clinical trials conducted over the past few decades, and multiple products have been approved for different diseases [64].

Animal studies have shown that MSCs can improve lung function in acute lung injury (ALI) mouse models and reduce the secretion of inflammatory cytokines [65]. Furthermore, preliminary observational studies suggest that MSCs may provide therapeutic benefits in COVID-19-related acute respiratory distress syndrome (ARDS) [66,67]. In severe or critical cases of COVID-19, ARDS remains a highly fatal condition that can result in pulmonary edema, arterial hypoxemia, and impaired lung function [68]. Unfortunately, treatment options for ARDS are still limited. However, many ongoing clinical trials involving over 1600 subjects have found the safety and efficacy of using MSCs in the treatment of severe COVID-19 cases [69,70,71,72,73,74,75,76,77].

Leng et al. found that treatment with an intravenous infusion of 1 × 106 umbilical cord mesenchymal stem cells (UC-MSCs)/kg for 14 days reduced inflammation and promoted lung tissue regeneration [76,78]. Sadeghi et al. found that MSC therapy improved oxygenation, cleared lung infiltrates, and reduced peripheral blood levels of inflammatory cytokines, with 80% of patients recovering and leaving the ICU within a median of 6 days after receiving one to two doses of intravenous MSCs [79]. Similar findings were reported by Lanzoni et al. [70,77]. In addition, a phase 2 clinical trial with a large number of patients showed that UC-MSC therapy significantly improved lung lesion volume on day 28. Data collected every 3 months by Shi et al. showed continued improvement in lung lesions at the 3-month mark in patients treated with UC-MSCs [72,73].

Clinical trial results have revealed that the use of MSCs for treating COVID-19 patients is not an unrealistic therapeutic approach. Furthermore, the use of MSC-based therapy is considered a suitable treatment option for severely ill COVID-19 patients [77,80,81]. However, further research is needed to evaluate the safety, efficacy, and long-term outcomes of using different types of MSCs [82]. Although clinical studies related to MSCs are still in their early stages, MSCs hold great promise as clinical tools for the future.

## 8. Discussion

**Figure 2:**
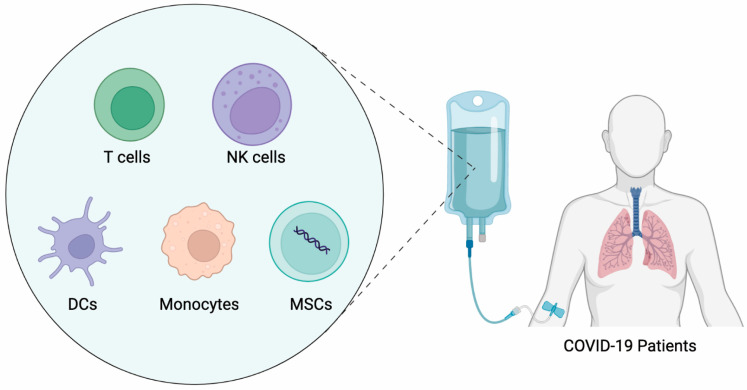
Different types of immune cell therapies for treating COVID-19.

Over the past years, immune cell therapy for cancer has continued to be enthusiastic, showing the great potential of immune cells for the treatment of human diseases. COVID-19 invasion has caused the concept of immune cell therapy to become highly popular once again. Several articles have focused on this field [83,84]. In this article, we summarized the types of cell therapies currently in the clinical trial stage (Figure 2) and listed the latest representative clinical trial programs for COVID-19 immune cell therapy (Table 1).

However, COVID-19 immune cell therapy is still in the early stages of development, and the key to its development will be the availability of high-quality immune cells and a standardized cell preparation process. The first key point is the high quality of immune cells. Research has confirmed that viral infections can lead to a decrease in the number of immune cells in the body, and even lead to a decline in cellular immune function [5]. Therefore, in order to carry out immune cell therapy, first, it is necessary to guarantee a sufficient number of these immune cells, especially for those therapies aimed at treating solid tissue diseases. Both cell migration and localization to the target site are important considerations, and these may depend heavily on the initial delivery route. Currently, other alternatives to intravenous administration are being investigated, including intradermal, intra-lymph node, subcutaneous, and others. These may provide solutions to this long-standing biological challenge. Secondly, it is important to ensure that these immune cells are of high quality. The efficacy of cell therapy depends heavily on the ability of the cells to maintain their functionality in vivo. Processes of in vitro cell pre-treatment, such as freezing and thawing, may have an impact on the function of the cells [85]. And, changes in the function of the living cells, such as depletion and lack of persistence, can occur after administration of the drug. The solutions to these challenges are often complex. It can be achieved through genetic modification methods, but also through pretreatment with cytokines, small molecules, hypoxia, and/or biomaterials in order to adjust their function to the desired application. The second key point is to have a standardized immune cell preparation process or production process. The development of closed automated production processes is a necessary and challenging task to produce safe, high-quality cell therapy products. Current areas of focus include manufacturing processes for cryopreservation, cell selection and cell activation, as well as automated batch monitoring for quality assurance purposes and the adoption of electronic recording systems. These interventions have the potential to reduce the heterogeneity of cell therapies and shorten production times. In the future, decentralized manufacturing processes will scale up the global availability of cell therapies, making them accessible to patients who are currently hard to reach.

Another avenue to consider for future immune cell therapy in COVID-19 is the use of a combination of immune cell therapies. For example, the synergistic effects of MSCs with other cell types, including lung endothelial cells and epithelial cells, have been studied [86]. These findings support the investigation of these two cell types as combination cell therapies.

Immune cell therapy is considered a highly active area of research because of the current positive clinical advances, and it is expected that more disruptive cell therapy products for the treatment of viral infections, such as COVID-19, will emerge in the near future.

## Figures and Tables

**Table 1:** Immune-cell-based therapy clinical trials for COVID-19.

Strategy	Study Title	Phase	NCT Number	Status
Specific T cell	Safety Infusion of Natural Killer cells or Memory T Cells as Adoptive Therapy in COVID-19 pneumonia or Lymphopenia	1 and 2	NCT04578210	Completed
	Generation of SARS-CoV-2-specific T Lymphocytes from Recovered Donors and Administration to High-risk COVID-19 Patients	1 and 2	NCT05447013	Recruiting
	Novel Adoptive Cellular Therapy With SARS-CoV-2 Specific T Cells in Patients with Severe COVID-19	1	NCT04351659	Recruiting
	Part Two of Novel Adoptive Cellular Therapy With SARS-CoV-2 Specific T Cells in Patients with Severe COVID-19	1 and 2	NCT04457726	Unknown status
	Viral Specific T Cell Therapy for COVID-19 Related Pneumonia	1	NCT04742595	Recruiting
Treg	REgulatory T Cell infuSion fOr Lung Injury Due to COVID-19 PnEumonia (RESOLVE)	1	NCT04468971	Completed
	RAPA-501-Allo Therapy of COVID-19-ARDS	1 and 2	NCT04482699	Terminated
	NLow Dose of IL-2 In Acute Respiratory DistrEss Syndrome Related to COVID-19 (LILIADE-COVID)	2	NCT04357444	Completed
	Tregs for the Treatment of Acute Respiratory Distress Syndrome (ARDS) Associated With COVID-19 (regARDS)	1	NCT05027815	Terminated
NK cells	A Phase I/II Study of Universal Off-the-shelf NKG2D-ACE2 CAR-NK Cells for Therapy of COVID-19	1 and 2	NCT04324996	Unknown status
	Fase I Clinical Trial on NK Cells for COVID-19	1	NCT04634370	Unknown status
	Natural Killer Cell (CYNK-001) Infusions in Adults With COVID-19	1 and 2	NCT04365101	Active, not recruiting
	Off-the-shelf NK Cells (KDS-1000) as Immunotherapy for COVID-19	1 and 2	NCT04797975	Withdrawn
	NK Cells Treatment for COVID-19	1	NCT04280224	Recruiting
Dendritic cells	Injection and infusion of LV-SMENP DC vaccine and antigen-specific CTLs	3	NCT04276896	Recruiting
	Phase I-II Trial of Dendritic Cell Vaccine to Prevent COVID-19 in Adults	1 and 2	NCT04386252	Withdrawn
	A Study to Evaluate the Efficacy, Immune Response, and Safety of a COVID-19 Vaccine in Adults ≥ 18 Years with a Pediatric Expansion in Adolescents (12 to <18 Years) at Risk for SARS-CoV-2	3	NCT04611802	Active, not recruiting
	Study to Describe the Safety, Tolerability, Immunogenicity, and Efficacy of RNA Vaccine Candidates Against COVID-19 in Healthy Individuals	3	NCT04368728	Completed
	A Study to Evaluate Efficacy, Safety, and Immunogenicity of mRNA-1273 Vaccine in Adults Aged 18 Years and Older to Prevent COVID-19	3	NCT04470427	Completed
	Dendritic Cell Vaccine to Prevent COVID-19	1	NCT04685603	Unknown status
	Dendritic Cell Vaccine, AV-COVID-19, to Prevent COVID-19 Infection	1	NCT04690387	Completed
	Phase I-II Trial of Dendritic Cell Vaccine to Prevent COVID-19 in Adults	1 and 2	NCT04386252	Withdrawn
	Preventive Dendritic Cell Vaccine, AV-COVID-19, in Subjects Not Actively Infected with COVID-19	2	NCT05007496	Completed
	Training the Innate Immune System Against SARS-CoV-2 (COVID-19) Using the Shingrix Vaccine in Nursing Home Residents (NH-Shingrix)	1	NCT04523246	Active, not recruiting
Monocytes	The MONACO Cell Therapy Study: Monocytes as an Anti-fibrotic Treatment After COVID-19 (MONACO)	1 and 2	NCT04805086	Unknown status
Mesenchymal stem cells	Mesenchymal Stem Cells Therapy in Patients With COVID-19 Pneumonia	Not applicable	NCT04713878	Completed
	A Proof of Concept Study for the DNA Repair Driven by the Mesenchymal Stem Cells in Critical COVID-19 Patients (REPAIR)	Not applicable	NCT04898088	Completed
	NestaCell^®^ Mesenchymal Stem Cell to Treat Patients with Severe COVID-19 Pneumonia (HOPE)	2	NCT04315987	Completed
	An Exploratory Study of ADR-001 in Patients with Severe Pneumonia Caused by SARS-CoV-2 Infection (COVID-19)	1	NCT04522986	Completed
	Therapeutic Study to Evaluate the Safety and Efficacy of DW-MSC in COVID-19 Patients (DW-MSC)	1	NCT04535856	Completed
	Mesenchymal Stromal Cells for the Treatment of SARS-CoV-2 Induced Acute Respiratory Failure (COVID-19 Disease)	1 and 2	NCT04345601	Completed
	Efficacy of Infusions of MSC from Wharton Jelly in the SARS-CoV-2 (COVID-19) Related Acute Respiratory Distress Syndrome (MSC-COVID19)	2	NCT04625738	Completed
	Mesenchymal Stem Cells for the Treatment of COVID-19	1	NCT04573270	Completed
	A Randomized, Double-Blind, Single Center, Efficacy and Safety Study of Allogeneic HB-adMSCs Against COVID-19.	2	NCT04348435	Completed
	A Clinical Trial to Determine the Safety and Efficacy of HB-adMSCs to Provide Protection Against COVID-19	2	NCT04349631	Completed
	A First-In-Human Phase 1b Study of AmnioPul-02 in COVID-19/Other LRTI	1	NCT05348772	Completed
	Menstrual Blood Stem Cells in Severe Covid-19	1 and 2	NCT05019287	Completed
	Treatment With Human Umbilical Cord-derived Mesenchymal Stem Cells for Severe Corona Virus Disease 2019 (COVID-19)	2	NCT04288102	Completed
	Use of UC-MSCs for COVID-19 Patients	1 and 2	NCT04355728	Completed
	Clinical Trial to Assess the Safety and Efficacy of Intravenous Administration of Allogeneic Adult Mesenchymal Stem Cells of Expanded Adipose Tissue in Patients with Severe Pneumonia Due to COVID-19	1 and 2	NCT04366323	Completed
	Treatment of COVID-19 Associated Pneumonia with Allogenic Pooled Olfactory Mucosa-derived Mesenchymal Stem Cells	1 and 2	NCT04382547	Completed
	The MEseNchymal coviD-19 Trial: MSCs in Adults with Respiratory Failure Due to COVID-19 or Another Underlying Cause (MEND)	1 and 2	NCT04537351	Completed
	Clinical Use of Stem Cells for the Treatment of Covid-19	1 and 2	NCT04392778	Completed
	Efficacy and Safety Evaluation of Mesenchymal Stem Cells for the Treatment of Patients with Respiratory Distress Due to COVID-19 (COVIDMES)	1 and 2	NCT04390139	Completed
	Evaluate the Safety and Efficacy of Allogeneic Umbilical Cord Mesenchymal Stem Cells in Patients With COVID-19 (UMSC01)	1 and 2	NCT05501418	Active, not recruiting
	Regenerative Medicine for COVID-19 and Flu-Elicited ARDS Using Lomecel-B (RECOVER) (RECOVER)	1	NCT04629105	Active, not recruiting
	Use of Mesenchymal Stem Cells in Acute Respiratory Distress Syndrome Caused by COVID-19	Early phase 1	NCT04456361	Active, not recruiting
	Multiple Dosing of Mesenchymal Stromal Cells in Patients with ARDS (COVID-19)	2	NCT04466098	Active, not recruiting
	Umbilical Cord Lining Stem Cells (ULSC) in Patients With COVID-19 ARDS (ULSC)	1 and 2	NCT04494386	Active, not recruiting
	Study of the Safety of Therapeutic Tx with Immunomodulatory MSC in Adults With COVID-19 Infection Requiring Mechanical Ventilation	1	NCT04397796	Active, not recruiting
	Efficacy and Safety Study of Allogeneic HB-adMSCs for the Treatment of COVID-19	2	NCT04362189	Terminated
	Study of Intravenous COVI-MSC for Treatment of COVID-19-Induced Acute Respiratory Distress	2	NCT04903327	Terminated
	hCT-MSCs for COVID19 ARDS	1 and 2	NCT04399889	Terminated
	MSCs in COVID-19 ARDS	3	NCT04371393	Terminated
	Mesenchymal Stem Cell Infusion for COVID-19 Infection	2	NCT04444271	Unknown status
	Mesenchymal Stem Cell for Acute Respiratory Distress Syndrome Due for COVID-19 (COVID-19)	2	NCT04416139	Unknown status
	Novel Coronavirus Induced Severe Pneumonia Treated by Dental Pulp Mesenchymal Stem Cells	Early phase 1	NCT04302519	Unknown status
	Safety and Efficacy of Mesenchymal Stem Cells in the Management of Severe COVID-19 Pneumonia (CELMA)	2	NCT04429763	Unknown status
	Safety and Effectiveness of Mesenchymal Stem Cells in the Treatment of Pneumonia of Coronavirus Disease 2019	Early phase 1	NCT04371601	Unknown status
	Mesenchymal Stem Cells in Patients Diagnosed With COVID-19	1	NCT04611256	Unknown status
	Bone Marrow-Derived Mesenchymal Stem Cell Treatment for Severe Patients with Coronavirus Disease 2019 (COVID-19)	1 and 2	NCT04346368	Unknown status
	Administration of Allogenic UC-MSCs as Adjuvant Therapy for Critically-Ill COVID-19 Patients	1	NCT04457609	Unknown status
	Mesenchymal Stem Cell Treatment for Pneumonia Patients Infected With COVID-19	1	NCT04252118	Unknown status
	Clinical Research of Human Mesenchymal Stem Cells in the Treatment of COVID-19 Pneumonia	1 and 2	NCT04339660	Unknown status
	Mesenchymal Stem Cell Therapy for SARS-CoV-2-related Acute Respiratory Distress Syndrome	2 and 3	NCT04366063	Unknown status
	Safety and Efficacy Study of Allogeneic Human Dental Pulp Mesenchymal Stem Cells to Treat Severe COVID-19 Patients	1 and 2	NCT04336254	Unknown status
	Treatment of COVID-19 Patients Using Wharton’s Jelly-Mesenchymal Stem Cells	1	NCT04313322	Unknown status
	Study of Human Umbilical Cord Mesenchymal Stem Cells in the Treatment of Severe COVID-19	Not applicable	NCT04273646	Unknown status
	Treatment of Coronavirus COVID-19 Pneumonia (Pathogen SARS-CoV-2) With Cryopreserved Allogeneic P_MMSCs and UC-MMSCs	1 and 2	NCT04461925	Unknown status
	Treatment of Severe COVID-19 Patients Using Secretome of Hypoxia-Mesenchymal Stem Cells in Indonesia	2	NCT04753476	Unknown status
	A Study of Cell Therapy in COVID-19 Subjects with Acute Kidney Injury Who Are Receiving Renal Replacement Therapy	1 and 2	NCT04445220	Unknown status
	A Study to Collect Bone Marrow for Process Development and Production of BM-MSC to Treat Severe COVID19 Pneumonitis (COMET20d)	Observational	NCT04397471	Unknown status
	Safety and Efficacy of Intravenous Wharton’s Jelly Derived Mesenchymal Stem Cells in Acute Respiratory Distress Syndrome Due to COVID 19	1 and 2	NCT04390152	Unknown status
	Umbilical Cord (UC)-Derived Mesenchymal Stem Cells (MSCs) Treatment for the 2019-novel Coronavirus(nCOV) Pneumonia	2	NCT04269525	Unknown status
	Mesenchymal Stromal Cells for the Treatment of Patients With COVID-19.	1 and 2	NCT05433298	Withdrawn
	ASC Therapy for Patients with Severe Respiratory COVID-19 (ASC COVID-19)	1 and 2	NCT04341610	Withdrawn
	Study of Allogeneic Adipose-Derived Mesenchymal Stem Cells to Treat Post COVID-19 “Long Haul” Pulmonary Compromise	2	NCT04909892	Withdrawn
	Study of Intravenous Administration of Allogeneic Adipose-Derived Mesenchymal Stem Cells for COVID-19-Induced Acute Respiratory Distress	2	NCT04728698	Withdrawn
	Study of Allogeneic Adipose-Derived Mesenchymal Stem Cells for Non-COVID-19 Acute Respiratory Distress Syndrome	2	NCT04909879	Withdrawn
	Umbilical Cord Tissue (UC) Derived Mesenchymal Stem Cells (MSCs) Versus Placebo to Treat Acute Pulmonary Inflammation Due to COVID-19 (COVID-19)	1	NCT04490486	Withdrawn
	BAttLe Against COVID-19 Using MesenchYmal Stromal Cells	2	NCT04348461	Suspended
	A Study of ADR-001 in Patients with Severe Pneumonia Caused by SARS-CoV-2 Infection (COVID-19)	2	NCT04888949	Recruiting
	A Clinical Study on Safety and Effectiveness of Mesenchymal Stem Cell Exosomes for the Treatment of COVID-19.	Early phase 1	NCT05787288	Recruiting
	Cord Blood-Derived Mesenchymal Stem Cells for the Treatment of COVID-19 Related Acute Respiratory Distress Syndrome	1 and 2	NCT04565665	Recruiting
	Application and Research of Mesenchymal Stem Cells in Alleviating Severe Development of COVID-19 Infection	1 and 2	NCT05741099	Recruiting
	UC-MSCs in the Treatment of Severe and Critical COVID-19 Patients	3	NCT05682586	Recruiting
	Allogenic UCMSCs as Adjuvant Therapy for Severe COVID-19 Patients (UCMSC)	2 and 3	NCT05132972	Recruiting
	A Phase II Study in Patients with Moderate to Severe ARDS Due to COVID-19	2	NCT04780685	Recruiting
	Study of Allogeneic Adipose-Derived Mesenchymal Stem Cells for Treatment of COVID-19 Acute Respiratory Distress	2	NCT04905836	Recruiting
	Randomized Double-Blind Phase 2 Study of Allogeneic HB-adMSCs for the Treatment of Chronic Post-COVID-19 Syndrome (HBPCOVID02)	2	NCT05126563	Recruiting
	Study to Evaluate the Efficacy and Safety of AstroStem-V in Treatment of COVID-19 Pneumonia	1 and 2	NCT04527224	Recruiting
	Safety and Efficacy of Umbilical Cord Mesenchymal Stem Cell Exosomes in Treating Chronic Cough After COVID-19	Early phase 1	NCT05808400	Recruiting
	UC-MSCs in the Treatment of Severe and Critical COVID-19 Patients with Refractory Hypoxia	3	NCT05689008	Recruiting
	Study of Descartes-30 in Acute Respiratory Distress Syndrome	1 and 2	NCT04524962	Recruiting
	Mesenchymal Stem Cells for the Treatment of Various Chronic and Acute Conditions	1 and 2	NCT04684602	Recruiting
	Repair of Acute Respiratory Distress Syndrome by Stromal Cell Administration (REALIST) (REALIST	1 and 2	NCT03042143	Recruiting
	Autologous Adipose-derived Stem Cells (AdMSCs) for COVID-19	2	NCT04428801	Not yet recruiting
	Mesenchymal Stromal Cells for COVID-19 and Viral Pneumonias (SAMPSON-1)	1	NCT05286255	Not yet recruiting
	Clinical Study for Subjects With COVID-19 Using Allogeneic Adipose Tissue-Derived Mesenchymal Stem Cells (AdMSCs)	2	NCT05017298	Not yet recruiting
	Treatment of Long COVID Symptoms Utilizing Autologous Stem Cells Following COVID-19 Infection	1	NCT05669261	Not yet recruiting
	Study of Allogeneic Adipose-Derived Mesenchymal Stem Cells to Treat Post COVID-19 “Long Haul” Pulmonary Compromise (BR)	2	NCT04992247	Not yet recruiting
	Efficacy and Safety of Umbilical Cord Mesenchymal Stem Cells in the Treatment of Long COVID-19	2	NCT05719012	Not yet recruiting
	Mesenchymal Stem Cells (MSCs) in Inflammation-Resolution Programs of Coronavirus Disease 2019 (COVID-19) Induced Acute Respiratory Distress Syndrome (ARDS)	2	NCT04377334	Not yet recruiting
	Use of hUC-MSC Product (BX-U001) for the Treatment of COVID-19 With ARDS	1 and 2	NCT04452097	Not yet recruiting
	AllogeneiC Expanded Human MSC Therapy in Patients Recovering From COVID-19 Acute Respiratory Distress Trial (ACE_CARD)	1	NCT05491681	Not yet recruiting
